# Congenital coenzyme Q5-linked pathology: causal genetic association, core phenotype, and molecular mechanism

**DOI:** 10.1007/s13353-023-00773-9

**Published:** 2023-08-21

**Authors:** Mateusz Dawidziuk, Aleksandra Podwysocka, Marta Jurek, Ewa Obersztyn, Monika Bekiesinska-Figatowska, Alicja Goszczanska-Ciuchta, Ewelina Bukowska-Olech, Agnieszka Magdalena Rygiel, Dorothy Lys Guilbride, Wojciech Wiszniewski, Pawel Gawlinski

**Affiliations:** 1 Department of Medical Genetics, Institute of Mother and Child, Warsaw, Poland; 2 Department of Diagnostic Imaging, Institute of Mother and Child, Warsaw, Poland; 3 Clinic of Neurology of Children and Adolescents, Institute of Mother and Child, Warsaw, Poland; 4 Department of Medical Genetics, Poznan University of Medical Sciences, Poznan, Poland; 5 Independent Researcher, Manhiça, Mozambique; 6 Department of Molecular and Medical Genetics, Oregon Health and Science University, Portland, USA

**Keywords:** COQ5, COQ10, Molecular mechanism, Expansion of the phenotype

## Abstract

Coenzyme Q5 (*COQ5*), a C-methyltransferase, modifies coenzyme Q10 (COQ10) during biosynthesis and interacts with polyA-tail regulating zinc-finger protein ZC3H14 in neural development. Here, we present a fifth patient (a third family) worldwide with neurodevelopmental and physiological symptoms including *COQ10* deficiency. Our patient harbors one novel c.681+1G>A and one recurrent p.Gly118Ser variant within *COQ5*. The patient’s mRNA profile reveals multiple *COQ5* splice-variants. Subsequently, we comprehensively described patient’s clinical features as compared to phenotype and symptoms of other known congenital coenzyme Q5-linked cases. A core spectrum of *COQ5*-associated symptoms includes reduced COQ10 levels, intellectual disability, encephalopathy, cerebellar ataxia, cerebellar atrophy speech regression/dysarthria, short stature, and developmental delays. Our patient additionally displays dysmorphia, microcephaly, and regressive social faculties. These results formally establish causal association of biallelic *COQ5* mutation with pathology, outline a core *COQ5*-linked phenotype, and identify mRNA mis-splicing as the molecular mechanism underlying all *COQ5* variant-linked pathology to date.

**Supplementary Information:**

The online version contains supplementary material available at 10.1007/s13353-023-00773-9.

## Introduction

*COQ5* is expressed in all human tissues (Nguyen et al. 2014) and affects fundamental developmental and cellular biology. As a protein, COQ5 interacts with poly-A regulating zinc-finger protein ZC3H14 affecting transcript stability, polypeptide levels in central nervous system (CNS) development (Pak et al. 2011; Najmabadi et al. 2011) and its methyl transferase activity provides essential modifications during biosynthesis of COQ10, a molecule crucial to mitochondrial and cellular eukaryotic metabolism (Nguyen et al. 2014; Yen et al. 2016; Hargreaves 2021).

Studies in yeast identified a minimum of 11 gene products (COQ1–11), including COQ5 which directly supports COQ10 biosynthesis. COQ1–9 form a multiprotein complex, the COQ synthome, found on the inner mitochondrial membrane (Nguyen et al. 2014; Yen et al. 2016; Hargreaves 2021). The COQ10 benzoquinone ring and 10-unit polyisoprenoid tail moieties synthesized in different subcellular locations are position together in the mitochondrion, where COQ5, 7, and 9 further modify COQ10. In addition, different COQ transcripts and polypeptides interact, interregulating stabilities, protein levels, and activities (Nguyen et al. 2014; Hargreaves 2021). These interactions impact both individual physiological COQ coenzyme functions not directly related to COQ10 biosynthesis (Hargreaves 2021), such as COQ5 interactions with polyA-tail regulating protein ZC3H14 during neural development (Pak et al. 2011), as well as COQ5 stability, influenced by COQ8, affecting COQ10 biosynthesis and COQ10-dependent metabolism (Nguyen et al. 2014; Yen et al. 2016; Hargreaves 2021).

Patients with identified biallelic mutation in any of the supporting COQ-enzymes (*COQ1–2, 4–9*) present with a range of developmental and physiological pathologies with a preponderance of neurodevelopmental manifestations and *COQ10* tissue deficiency (Yen et al. 2016; Hargreaves 2021; Malicdan et al. 2018; Online Mendelian inheritance in man n.d.). These are defined as primary *COQ10* deficiencies. Indirect factors causing a *COQ10* deficiency such as aging, disease or certain drugs, resulting in malabsorption, or disrupted bio-distribution, for example, are secondary. Nonetheless, some symptomatic patients with biallelic mutations have normal COQ10 levels in tissues tested, indicating that their condition is not a straightforward related to COQ10 deficiency; others with established COQ10 deficits do not respond to oral COQ10 supplementation, suggesting involvement of additional factors.

Correlation of symptomology with a molecular basis of COQ10 biosynthesis/deficiency is clinically and therapeutically highly relevant. For example, neurodevelopmental defects associated with *COQ10* deficiency due to a biallelic *COQ10* disruption are mostly effectively treated with appropriate oral COQ10 supplementation, particularly if identified early in childhood. COQ5 mutations (Malicdan et al. 2018), which destabilizes the COQ5 polypeptide, leading to disrupted COQ10 biosynthesis, cause COQ10 deficiency that respond well to supplementation, within weeks. Accurate and rapid differential molecular diagnoses are crucial for treatment of clinically overlapping syndromes and enable for proper prognosis, planning, therapeutic measures, and quality of life, for both patient and family.

Here, we identified a novel *COQ5* pathogenic variant and investigated the molecular mechanism underlying its pathology. We also characterized a specific constellation of clinical traits linked to mutations at the *COQ5* locus, identified so far.

## Materials and methods

RNA extraction, cDNA synthesis, PCR, cloning, and exome sequencing were done according to manufacturer’s instructions. For more details, please see supplementary data.

## Results

### Patient history

We present a Polish female patient current age 10 years, born at term by natural delivery at the 39th week of the mother’s first pregnancy. Parents were healthy, non-consanguineous, Caucasian in origin. At the time of birth, the mother was 29 years old; the father was 33 years old.

Family history was negative for individuals with neurodevelopmental disorders. Patient birth body parameters were the following: body weight: 3380 g (50 pc); body length: 54 cm (<95 pc); occipital frontal circumference: 31 cm (<3 pc). Apgar scores were 10 points at 1 and 5 min of life.

Presented girl displayed normal, uneventful postnatal adaptation and development from birth to 5 months of life.

At 22 weeks, directly after third dose-vaccination with DTaP (Infanrix-Hexa), motor and cognitive regression, deterioration of eye contact, and the lack of a smile were noticed. Consequently, further vaccinations were suspended. At the age of 18 months, further developmental regression became apparent. She was intensively rehabilitated, but no progress was observed. In clinical evaluation, she could not stand up or walk independently and she could pronounce a few simple syllables and try to combine them into words. Detailed clinical observations from 1.5 to 10 years of age are described in supplementary data.

**Fig. 1:**
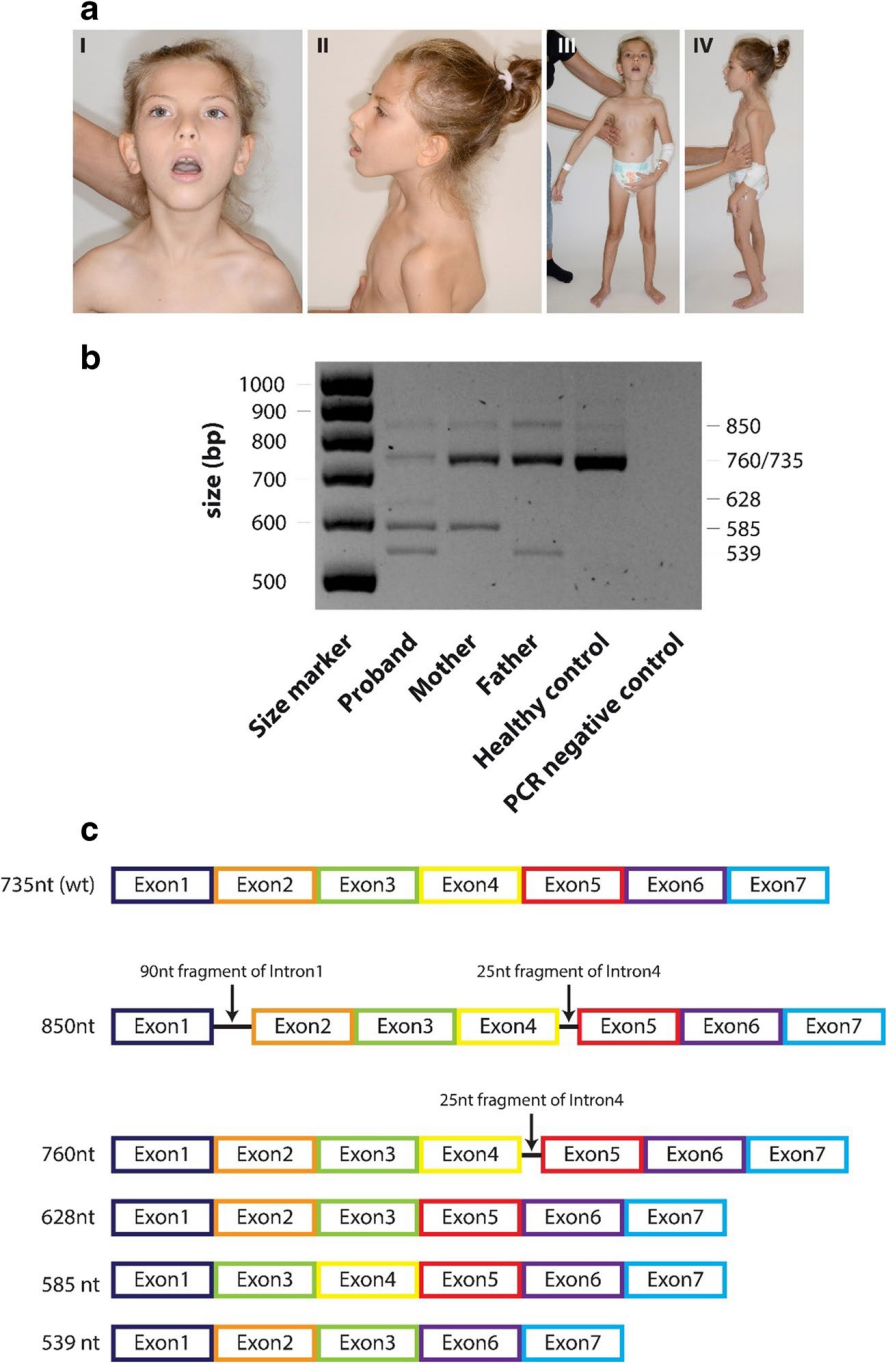
**a** Photographic documentation of the patient at age 10 years old. **b** *COQ5* cDNA gel electrophoresis. **c** Graphical representation of the splicing products of the *COQ5* gene in a patient, her parents, and healthy control. For more details, please see supplementary data

At the age of 10 years, she had not acquired the ability to walk; cognitive development remains at a low level. Summary of neurological examinations showed global developmental delay, tetraparesis with hypotonia and symmetrical tendon reflexes, limited eye contact, postural defects including a “rounded” back, protruding shoulder blades, and funnel-shaped chest (Fig. 1aIII–IV). Phenotypic evaluation of the face revealed slight ptosis, a tendency to open the mouth and tilt the head back, without other specific features of dysmorphism (Fig. 1aI).

### Exploratory assays

Magnetic resonance imaging (MRI) of the brain at the age of 8 years revealed clear cortico-subcortical atrophy of the cerebellum–enlargement of the 4th ventricle and widening of cerebellar sulci (Supp.Fig.1a-d, arrows) as compared to the normal appearance in a healthy individual (Supp.Fig.1e-h, arrows). The cerebellar sulci were wider in our patient at age 8 years than at 18 months, as well as in comparison to an age-matched healthy control. This indicates progressive atrophy of cerebellar tissue. Atrophy involved both vermis and cerebellar hemispheres. The supratentorial structures are normal. Echocardiogram, abdominal ultrasonography, and ophthalmologic investigation were also uneventful.

Low COQ10 level tests showing 0.6 mg/l (normal range >0.67 mg/l) and corroborated twice at age 9 after 2 months of irregular QuinoMit 32.5 mg/day nasal spray administration, level of COQ10 in leukocytes remained low (Table 1 p.55–56). For the last 1.5 years, she has been treated with QuinoMit Q10 Fluid at a dose of 26 mg every second day, with good tolerance. We are observing a gradual improvement in the patient’s motor condition—muscle strength is better, the patient sits up on her own from a lying position, stands on her own, and the ptosis has decreased. However, she has not acquired the ability to walk; cognitive development remains at a low level.

**Table 1:** Clinical features for *COQ5* patient 5 compared to reported symptomology for 4 previous *COQ5* patients

#	Features	Najmabadi et al. (2011)	Malicdan et al. (2018)			Present report
	Patient	Patient 1	Patient 2 (III.4)	Patient 3 (III.3)	Patient 4 (III.6)	Patient 5
	Mutation	c.352G>A p.(Gly118Ser)hom	c.575-1761_*1489dup p.(?)	c.575-1761_*1489dup p.(?)	c.575-1761_*1489dup p.(?)	c.352G>A/681+1G>A p.Gly118Ser/?
	Gender	nd	Female	Female	Female	Female
	Current age	nd	nd	nd	nd	10 years
	Parental consanguinity	Yes	No	No	No	No
	Ethnicity	nd	Iraqi-Jewish	Iraqi-Jewish	Iraqi-Jewish	Polish
1	Encephalomyopathy	nd	+	+	+	−
2	Cerebellar ataxia (non-progressive)	nd	+	+ (mild)	+	+
3	Encephalopathy	nd	+	+	+	+
4	Generalized tonic clonic seizures	nd	+ (17 years old several)	+ (22 years old single)	+	−
5	Developmental delay	nd	+	+	+	+
6	Short stature	nd	+	nd	+	+ (−3.19 SDS 9 years old)
7	Delay in motor milestones development	nd	+ (moderate)	nd	+ (mild)	+
8	Delay in cognitive milestones	nd	+ (moderate)	nd	nd	+
9	Dysarthria	nd	+	+ (mild)	+ (mild)	na (lack of speech)
10	Mild-moderate cognitive disability	nd	+	below average intelligence	nd	regression after 6 months
Behavioral problems						
11	Impulsivity	nd	+	nd	nd	+
12	Attention deficiency	nd	+	nd	nd	+
13	Oppositional defiant disorder	nd	+	nd	nd	−
14	Myoclonic jerks	nd	+ (12 years old)	+ (20 years old)	nd	−
15	Epilepsy	nd	+	nd	nd	−
16	Microcephaly	nd	−	−	−	+ (−3.56 SDS 9 years old)
17	Nystagmus	nd	+	+ (horizontal)	+ (horizontal)	−
18	Slow saccades	nd	+	nd	nd	−
19	Saccadic movements	nd	+	nd	+	−
20	Apraxic gaze	nd	+	nd	nd	−
21	Fundoscopy	nd	normal	nd	nd	−
22	Dysarthric cerebellar speech	nd	+	nd	nd	na (lack of speech)
23	Dysmetria in finger-to-nose test	nd	+	nd	−	−
24	Postural and intention tremor	nd	+	nd	−	−
25	Ataxic gait	nd	+	+ (mild)	nd	na (lack of gait)
26	Dysmetria and oculomotor apraxia	nd		+ (mild)	nd	−
27	Abnormal gait pattern	nd	+	+	abnormal tandem walking identified at 14 years old	na (lack of gait)
28	Lower limb spasticity	nd	+ (mild)	nd	+	+
29	Cerebellar atrophy	nd	+ (non-progressive)	+ (mild)	nd	+
30	EEG	nd	infrequent generalized polyspike-wave discharges	infrequent generalized polyspike-wave discharges	nd	normal
31	Nerve conduction study	nd	normal	nd	nd	nd
32	Cardiac echocardiogram	nd	normal	nd	nd	normal
Morphology and biochemistry						
33	Complete blood count	nd	normal	nd	nd	normal
34	Electrolyte levels	nd	normal	nd	nd	normal
35	Creatine phosphokinase	nd	normal	nd	nd	normal
36	Liver and renal function tests	nd	normal	nd	nd	normal
37	Carnitine and acyl-carnitine	nd	normal	nd	nd	normal
38	Copper	nd	normal	nd	nd	normal
39	Ceruloplasmin	nd	normal	nd	nd	nd
40	Thyroid function tests	nd	normal	nd	nd	normal
41	Lactate	nd	normal	nd	nd	normal
42	Pyruvate	nd	normal	nd	nd	nd
43	Ammonia	nd	normal	nd	nd	nd
44	Blood amino acid profile	nd	normal	nd	nd	normal
45	Very long chain fatty acids	nd	normal	nd	nd	nd
46	Phytanic acid	nd	normal	nd	nd	nd
47	Homocysteine	nd	normal	nd	nd	normal
48	Isoelectric focusing of transferrin	nd	normal	nd	nd	nd
49	Alpha-fetoprotein	nd	normal	nd	nd	nd
50	Quantitative immunoglobulin levels	nd	normal	nd	nd	nd
51	Vitamin E levels	nd	normal	nd	nd	nd
52	Urine for protein	nd	normal	nd	nd	normal
53	Urine for organic acids	nd	normal	nd	nd	normal
54	Dysmorphia	nd	−	nd	nd	+
55	COQ10 enzyme tests values in leukocytes before COQ treatment	nd	65 pmol/μg*	78 pmol/μg*	72 pmol/μg*	0.6 mg/l**
56	COQ10 enzyme tests values in leukocytes after COQ treatment	nd	after 3-month treatment: 293 pmol/μg*	after 3-month treatment: 332 pmol/μg*	after 3-month treatment: 279 pmol/μg*	after 2-month treatment: 0.7 mg/l**
58	Other biochemical tests abnormalities	nd	III.4	III.3	III.6	nd

*nd* no data, *na* not applicable

*Normal range 119.86 ± 24.23

**Normal range > 0.67 mg/l

### Molecular results

The aCGH analysis did not show any pathogenic copy number variations. Exome sequencing (ES) revealed one novel variant c.681+1G>A and one recurrent pathogenic variant c.352G>A (p.Gly118Ser) in *COQ5* (Najmabadi et al. 2011). Biallelic origin was confirmed by analysis of the patient’s parents using Sanger sequencing (Supp.Fig.2c). Examination of *COQ5* mRNA (Supp.Fig.3) showed that its level is markedly diminished in our patient (Supp.Fig.3 band 735 bp compared to the mRNA band in mother, father, and healthy control). Mis-spliced RNA forms in the patient indicate specific maternal (Supp.Fig.3 band 585 bp) and paternal (Supp.Fig.3 band 539 bp) mis-splice contributions. Exons, ribonucleotide sequence, and amino acid sequence for each band depicting mis-splices are shown in Supp.Fig.3. The patient also harbors a mis-splice that did not contain exon 4 (Supp.Fig.3, band 628 nt) undetected in parents and healthy control.

### COQ5-linked syndrome phenotype

The available genetic and clinical information reported for all known patients with *COQ5* mutations compared with our patient 5 is compiled in Table 1. This provides a core phenotype (Table 1: 2, 3, 5, 6, 9, 10, 29, 55) linked to *COQ5* disruption.

## Discussion

In this study, we identified a novel splicing c.681+1G>A *COQ*5 variant. Up to date, this is a third pathogenic variant found at the *COQ5* locus. The variant was detected in the patient harboring known pathogenic p.Gly118Ser *COQ5* variant on the other allele. Biallelic origin of the variants and recessive mode of inheritance was confirmed by Sanger sequencing of parent’s DNA. The patient’s mRNA profile indicated that both inherited *COQ5* variants contribute to characteristic mis-spliced mRNA forms, deleting either exon 2, or exons 4 and 5. Further, we comprehensively described clinical features of our patient as compared to phenotype and symptoms of other known congenital coenzyme Q5-linked cases. These allowed us indicating a specific constellation of clinical traits linked to mutations at the *COQ5* locus.

Our patient has *COQ10* deficiency and pronounced neurodevelopmental traits. Evidently, neither *COQ5* variant supports normal *COQ5* function or healthy COQ10 levels. If either did, our patient would show healthy neurodevelopment with normal *COQ10* levels. Each of these two variants therefore, directly confirms association of the other with pathology. Which symptoms in our patient result from COQ10 deficiency, versus other COQ5-associated enzymatic, regulatory, or structurally associated dysfunctions, such as binding interactions with CZ3H14, or COQ8, or the COQ-synthome itself, are elusive.

Compilation of the phenotypes for all five biallelic *COQ5* patients (Table 1), exposes common traits presenting together in each patient and which as a set, are largely non-overlapping with other COQ protein-associated pathologies (Online Mendelian inheritance in man n.d.). This suggests a specific constellation of clinical traits linked to mutations at the COQ5 locus, so far, encompassing cerebellar ataxia, encephalopathy, developmental delay, short stature, dysarthria, ID, cerebellar atrophy, and COQ10 deficiency in leucocyte assay (Table 1: 2, 3, 5, 6, 9, 10, 29, 55, respectively). Although each trait may occur individually in many unrelated neurodevelopmental disorders, and also in patient pathology profiles with other *COQ* loci biallelic mutation, we propose that a majority (5/8 or more) of these traits occurring together represents a core phenotype linked to *COQ5* disruption. While not diagnostically definitive, a clinical perspective pointing strongly to a specific *COQ*-locus, in this case *COQ5*, should accelerate the diagnostic procedure and enable crucial interventions earlier in patient development. Eventually, gene-trait associations should allow mapping of specific traits to protein domains, specific functions, and particular alleles, informing etiologic gene test panels. These could facilitate rapid differential clinical diagnoses for specific traits, syndromes, or compound conditions, early on in developmental stages, potentially even in utero. For example, rapid in utero distinction of primary versus secondary *COQ10* deficiencies in biallelic patients, eliminating the “trial and error” factor currently inherent in both diagnosis and *COQ10* supplementation therapy, could be life-changing.

In conclusion, our data clarifies the intricate molecular biology underlying *COQ*-locus clinical pathologies and COQ10-related metabolism, with concomitant progress in clinical handling.

## Supplementary information

ESM 1 (DOCX 644 kb)
