# Supplementary material for: Congenital coenzyme Q5-linked pathology: causal genetic association, core phenotype, and molecular mechanism
Source: J Appl Genet. 2023 Aug 21;64(3):507–14. doi: 10.1007/s13353-023-00773-9 (PMC10457220; doi:10.1007/s13353-023-00773-9)
Supplement: Supplementary file 1 — (DOCX 644 kb) [file 13353_2023_773_MOESM1_ESM.docx]

**Supplementary data:**

**Patient history**

We present a Polish female patient current age 10y, born at term by natural delivery at the 39th week of the mother’s first pregnancy. During pregnancy she developed gestational diabetes and diet treatment was necessary, as well as antispasmodic drugs because of premature contractions at 36th week. Parents are non-consanguineous, Caucasian in origin. At the time of birth the mother was 29 years old and was treated because of Hashimoto disease. The father was 33 years old and healthy. Family history was negative for individuals with neurodevelopmental disorders except of the mother’s cousin who had a son with Down syndrome and in in more distant relatives a case of hydrocephalus and cerebral palsy. Patient birth body parameters were the following: body weight: 3380g (50 pc); body length: 54 cm (< 95pc), occipital frontal circumference (OFC): 31cm (<3 pc). Apgar scores were 10 points at 1 and 5 min of life respectively.

Presented girl displayed normal, uneventful postnatal adaptation and development from birth to 5 months of life. In mothers’ relation she made eye contact, smiled, turned over and raised herself up on her hands in a prone position to crawl. She could held a toy in her hand, but she couldn't reach for the toy or lower limbs.

At 5 month and 2 weeks, directly after third dose-vaccination with DTaP (Infanrix-Hexa), motor and cognitive regression, deterioration of eye contact, the lack of a smile were noticed. Because of it further vaccinations were suspended.

Due to lack of progress in development, to improved postural control, motor and cognitive development, rehabilitation initially using the Vojta and later other different methods (e.g. Bobath’s) was started at the age of 9,5 months and its continues to this day.

**Clinical observations from 1.5 years to 10 years of age:**

At the age of 18 months further developmental regression became apparent. The girl was intensively rehabilitated, but no progress was observed. In clinical evaluation she couldn’t stand up or walk independently and she could pronounce a few simple syllables and try combine them into words.

At the age of 3y she started sitting up on her own. There was no progress in development until age 8.5y. Neurological examination at this time, indicates that the patient was conscious, made limited eye contact, had no verbal-logical contact, did not follow instructions and made stereotypical arm movements. The mother points out that the girl's cognitive and speech development alternates between periods of progress and regression. In the period of more intensive motor rehabilitation, the progress in speech development slows down.

At the age of 9y she can stand upright, but without possibility of locomotion .

At the age of 10y, she has not acquired the ability to walk, cognitive development remains at a low level. Until now she had not developed tweezer-grip but she can pointed with the whole hand and ate with her hands. Despite very intensive stimulation speech development significantly impaired. She pronouncing only few single syllables and inarticulate sounds. Behavior abnormalities with outbursts of screaming are quite common at present time. Additionally she does not control physiological needs.

Summary of neurological examinations at age 10y showed: global developmental delay, tetraparesis with hypotonia and symmetrical tendon reflexes, limited eye contact, postural defects include a "rounded" back, protruding shoulder blades and funnel-shaped chest (Fig 1a III-IV).

No tremors or dysmetry nor further pathological symptoms were observed. Cranial nerves were otherwise unchanged. EEG in wakefulness was without any noticeable changes and nerve conduction study were also normal.

Anthropometric body evaluation at 8,5y showed delayed somatic development with: primary microcephaly (head circumference 47.5 cm: -3,56 SD), low body weight (15,6 kg: -2,17 SD) and short stature (114.5 cm: -3,19 SD), BMI: 11.9: -1,68SD.

Phenotypic evaluation of the face showed the presence of a slight ptosis, a tendency to open the mouth and tilt the head back, without other specific features of dysmorphism (Fig. 1a I).

**Exploratory assays**

Specific genetic panel screening excludes Congenital Rett, Rett-like and Angelman's syndromes and genetically based lysosomal diseases. Specific genetic panel screening excludes: congenital Rett, Rett-like and Angelman's syndromes and microdeletion / microduplication syndromes, lysosomal diseases.

At 18 months, computed tomography (CT) of the brain, under general anesthesia, was within normal limits.

Immediately post-CT anesthesia at 1.5 years, further developmental regression became apparent.

General physiological parameter laboratory tests at age 8,5y showed slightly low red cell counts and normal ferritin concentration with no other significant deviations in physiological parameters. Metabolic tests for carnitine and acylcarnitine and amino acid profile in a dry blood drop using the tandem MS method were normal. GC/MS urinary organic acids profile and profile of transferrin isoform were normal too. Plasma aminogram was slightly above normal (alanine). Additionally: electrolyte levelsm creatine phosphatase, liver and renal function tests, Cooper, ceruloplasmine, thyroid hormone were in normal range. Lactic acid levels were slightly elevated in two successive tests: 2.43 (0.5-2.2) [mmol/L] and 2.91 (0.5-2.2) [mmol/L]. Echocardiogram and abdomen ultrasonography were also normal. (Table 1, patient 5). A detailed list of all traits and developmental observations for our patient is given in Table 1.

**Materials and Methods**

**RNA extraction and cDNA synthesis**

RNA was extracted from 2.5 ml of whole blood using PAXgene Blood RNA Kit IVD (PreAnalytiX) with manual RNA purification according to manufacturer protocol. RNA concentration was measured using standard RNA measurement protocol on DS-11 FX+ Spectrophotometer (DeNovix). cDNA synthesis used High-Capacity cDNA Reverse Transcription Kit (Applied Biosystems) with random hexamer primers according to manufacturer protocol. RNA samples were normalized to 500 ng/µl prior to cDNA synthesis.

**PCR, primers and product visualization**

PCR was carried out with Taq DNA polymerase in 10X Taq Buffer with (NH_4_)_2_SO_4_ (Thermo Scientific) on the MasterCycler Nexus GX2 thermocycler (Eppendorf) at 95°C for 5 minutes; 35 cycles at 95°C for 30 seconds, 60°C for 30 seconds, 72°C for 60 seconds; and 72°C for 10 minutes. Primer sequences: Forward: COQ5-F: 5’-GGTTTGAGACTGTGTCGGA-3’,

Reverse: COQ5-R: 5’-TCTCAGGAAGAGTTCAAGGACA-3’.

PCR cDNA products were separated on a 1.5% agarose gel run at 100 V for one hour in TBE buffer and visualized using an Gel Doc XR+ Gel Documentation System (Bio-Rad).

**PCR cloning and sequencing**

Gel-purified PCR products (PureLink PCR Purification Kit, Invitrogen) were subcloned (QIAGEN PCR Cloning Kit QIAGEN) into Subcloning Efficiency DH5α Competent Cells, Invitrogen. Briefly: 100ng of PCR product (10X molar excess for ~700bp PCR product) was added to a ligation-reaction mixture according to manufacturer protocol and left at 4^o^C overnight. Then 5 μl of ligation-reaction mixture were transfected into 50 μl DH5α competent cells, which were kept on ice for 30 minutes, then heat-shocked (42°C/20 seconds) and placed on ice again (2 minutes). 950 ml of LB medium were added and incubated for 1 hour at 37°C/300RPM. Bacteria were plated overnight at 37°C on selective medium (LB agarose, with 100 μg/ml of ampicillin). Picked colonies were cultured overnight in 3 ml LB with 100 μg/ml of ampicillin. Plasmid DNA was isolated with Plasmid Midi AX kit (A&A Biotechnology). Purified plasmid DNA was amplified by the means of PCR using M13 primers, visualized on 1.5% agarose gel run at 100 V. PCR products containing cDNA fragments of different length were sequenced using BigDye Terminator v3.1 Cycle Sequencing Kit (Applied Biosystems) on ABI 3500 Genetic Analyzer sequencer (Applied Biosystems).

**Exome sequencing (ES), bioinformatics pipeline and variant prioritization**

Genomic DNA was extracted from EDTA-treated blood samples using an automated chemagic 360 Instrument (Perkin Elmer). ES was performed for proband only, targeted Sanger sequencing was used for cosegregation analysis of selected variants in proband and biological parents. For ES we used Twist Human Core Exome Kit (Twist Bioscience) on a Novaseq 6000 (Illumina) platform with 100bp paired-end sequencing.

Data was processed with in-house pipeline. Raw sequencing reads were aligned to the GRCh38/hg38 assembly using the BWA v0.7.17 [9] with default parameters. GATK v4.1.4.0 [10] was used for insertion/deletion realignment and base quality score recalibration (BQSR). Single nucleotide variants (SNVs) and short insertion/deletion (INDELs) were identified using GATK HaplotypeCaller. Hard filtering used two possible filters for SNVs and INDELs, respectively: SNP filter: QD (Quality of Depth) < 2.0, MQ (Mapping Quality) < 30.0, MQRankSum < -12.5, ReadPosRankSum < -8.0 and FS > 60; INDEL filter: QD < 2.0, FS > 200, SOR > 10 and ReadPosRankSum < -20.0. Quality control was carried out with FastQC v0.11.7 [11], Samtools v1.9 [12], QualiMap v2.2.2c [13] and Bcftools v1.9 [12]. MultiQC v1.7 [14] generated quality report from listed programs. Prior to annotation variants were decomposed and normalized using vt v0.57721 [15]. Finally, resulting variants were annotated using Ensemble Variant Effect Predictor (VEP, release 107) [16] including annotations from different databases: genome Aggregation Database (gnomAD v2.1.1 liftover and v3.1.2) [17], Online Mendelian Inheritance in Man (OMIM; accessed 2022.11.20) [18], ClinVar (accessed 2022.11.13) [19], dbNSFP v4.2a [20], SpliceAI [21] and an in-house variant database consisting of 2109 exome samples from Polish population. 173.8 M reads were generated, 99.9% properly mapped, of which 62.5% were on-target unique mapped reads. Average coverage was 174x with 99.2% of the target DNA covered at least 20x. Variant calling produced 205126 variants; 174650 SNV, 14671 insertions and 15805 deletions. Variant prioritization were as follows: i. Variants reported as pathogenic and/or probably pathogenic in the ClinVar database were screened for clinical relevance in relation to the patient's phenotype, zygosity of the variant and mode of inheritance of the corresponding disease. ii. Based on patient clinical characteristics, an in silico panel of genes were created using selected HPO database terms [22]: global developmental delay (HP:0001263), microcephaly (HP:0000252), hypotonia (HP:0001252), absent speech (HP:0001344), developmental regression (HP:0002376). HPO database accessed 2022.11.23. In further analyses, variants present in genes of this panel were filtered as follows: only variants passing the quality filter (PASS); below 0.001 frequency in the gnomAD v3.1.2 database and in house variant database; variants reported in the ClinVar database as benign and/or probably benign were discarded; variants only in coding regions (including splice intron variants and synonymous variants within splice sites); zygosity consistent with mode of inheritance of corresponding disease; in diseases where significant proportion of clinical findings fit patient phenotype. All resulting variants were classified following American College of Medical Genetics and Genomics (ACMG) criteria [23].


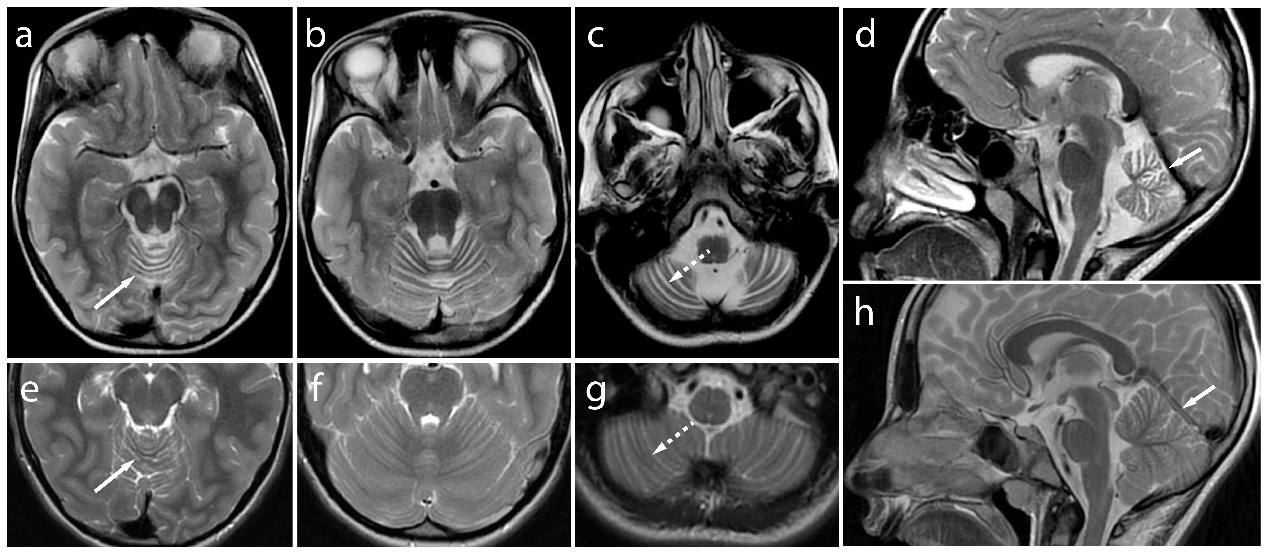


Supplementary Figure 1. Corresponding axial sections of magnetic resonance imaging (MRI) at age 8y (**a**-**c**): atrophy affecting vermis (**a**, arrow), and cerebellar hemispheres (**c**, dotted arrow). **e**-**g:** healthy age-matched control showing normal width of vermian (**e**, arrow) and hemispheric sulci (**g**, dotted arrow). **d**,**h**: Additional sagittal sections of MRI show the full extent of vermian atrophy (arrows): (**d**) patient’s vermis; (**h)** healthy age matched vermis.


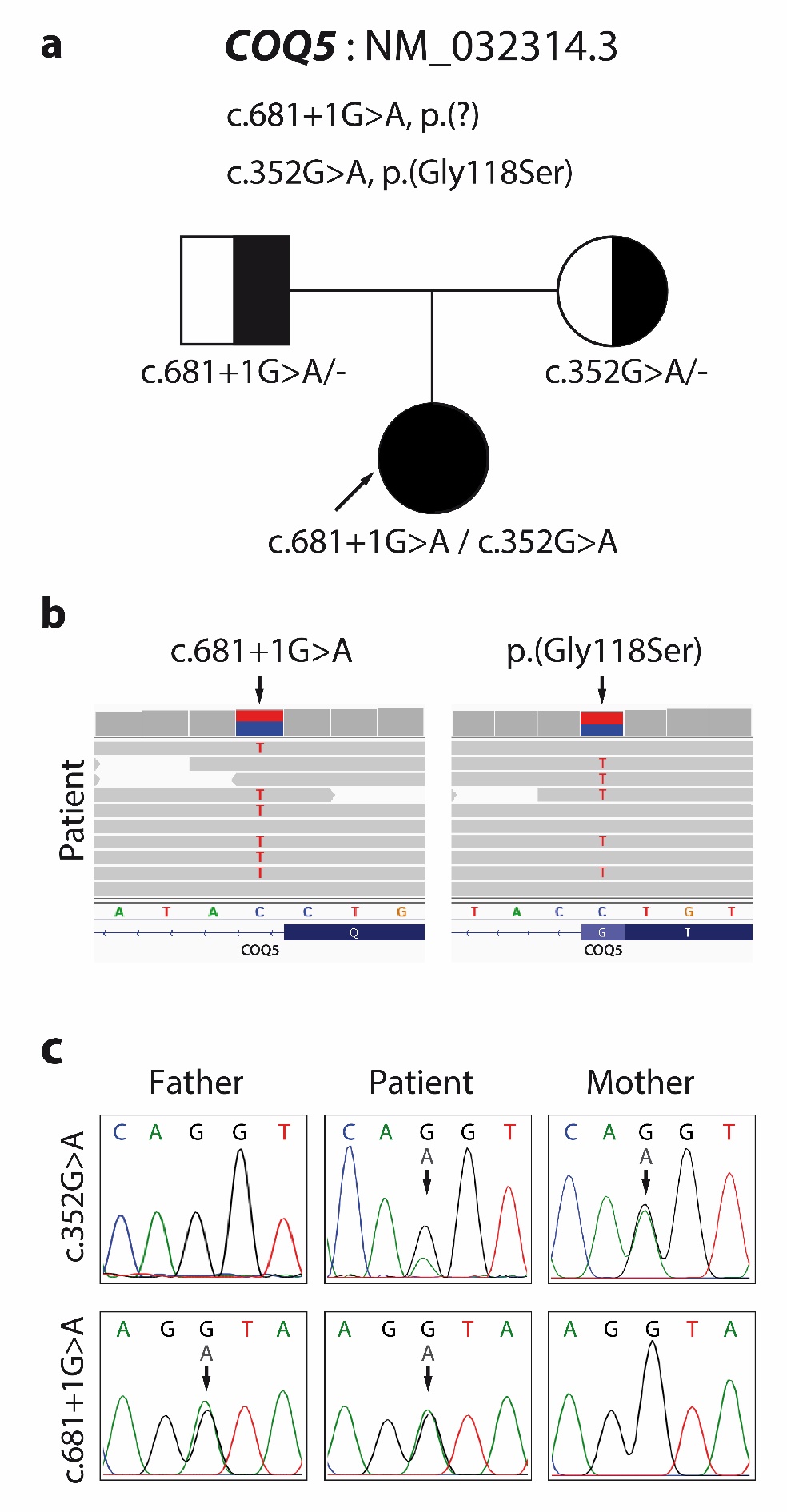


Supplementary Figure 2. Pedigree (**a**), visual presentation of exome sequencing data in the Integrative Genomics Viewer (**b**) and Sanger sequencing (**c**) confirming biparental character of inherited mutations c.681+1G>A / c.352G>A (NM_032314.4; NP_115690.3; human genome version GRCh38) in *COQ5* in patient 5.


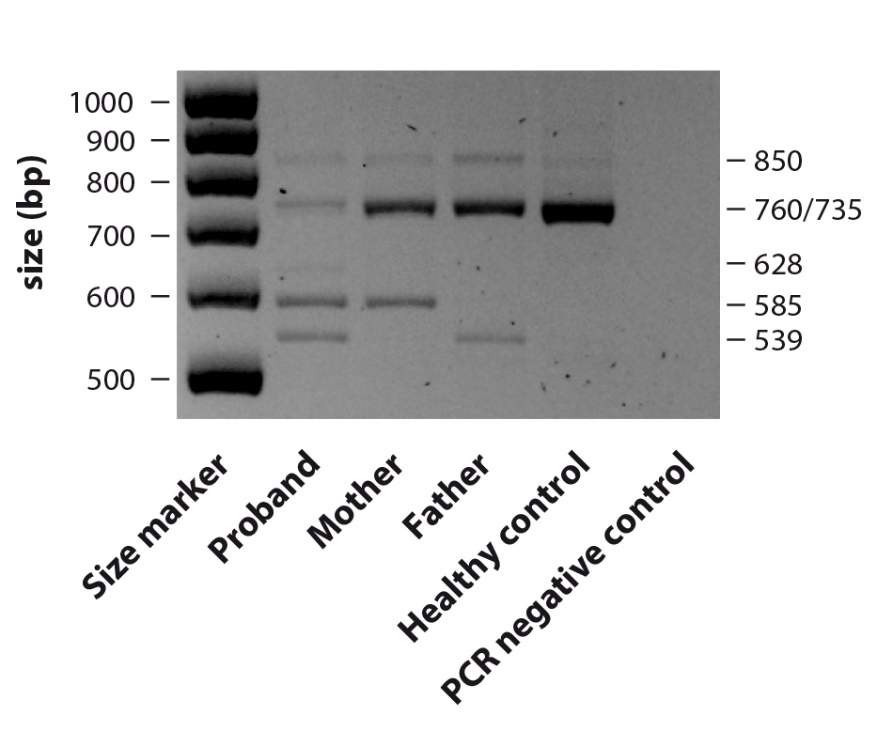


**Band 850 bp** – cloned and sequenced fragment of *COQ5* mRNA contained additional fragment of intron 1 (small letters, black, italics, length 90 bp) between exon 1 and 2, and additional fragment of intron 4 (length 25bp) between exon 4 and 5:

**Exon1**

**GGTTTGAGACTGTGTCGGAAGAGGAGAAGGGGGGCAAAG*aactcctttaatgtgaaagtttt***

**G--F--E--T--V--S--E--E--E--K--G--G--K--E--L--L--*--**

***Fragment of Intron1 (90 bp)***

***tgctggcatcacttcttctgggacatcttcaacctttttgtcacagctccatttcttgtt***

***catgttg*TCTATCAGGTGTTTGAAAGTGTGGCTAAGAAGTATGATGTGATGAATGATATG**

**Exon2**

**ATGAGTCTTGGTATCCATCGTGTTTGGAAGGATTTGCTGCTCTGGAAGATGCACCCGCTT**

**CCTGGGACCCAGCTGCTTGATGTTGCTGGAGGCACAGGTGACATTGCATTCCGGTTCCTT**

**AATTATGTTCAGTCCCAGCATCAGAGAAAACAGAAGAGGCAGTTAAGGGCCCAACAAGAT**

**Exon3**

**TTATCCTGGGAAGAAATTGCCAAAGAGTACCAGAATGAAGAAGATTCCTTGGGCGGGTCT**

**CGTGTCGTGGTGTGTGACATCAACAAGGAGATGCTAAAGGTTGGAAAGCAGAAAGCCTTG**

**GCTCAAGGATACAGAGCTGGACTTGCATGGGTATTAGGAGATGCTGAAGAACTGCCCTTT**

**Exon4**

**GATGATGACAAGTTTGATATTTACACCATTGCCTTTGGGATCCGGAATGTCACACACATT**

***Fragment of Intron4 (25bp)***

**GATCAG*atatgaatggctgcatcctcagctt*GCACTCCAGGAAGCTCATCGGGTGCTGAA**

**Exon5**

**ACCAGGAGGACGGTTTCTCTGTCTGGAATTTAGCCAAGTGAACAATCCCCTCATATCCAG**

**GCTTTATGATCTATATAGCTTCCAGGTCATCCCTGTCCTGGGAGAGGTCATCGCTGGAGA**

**Exon6 Exon7**

**CTGGAAGTCCTATCAGTACCTTGTAGAGAGTATCCGAAGGTTTCCGTCTCAGGAAGAGTT**

**CAAGGACA**

**Band 760 nt** - cloned and sequenced fragment of *COQ5* mRNA contained additional fragment of intron 4 (small letters, black, italics, length 25 bp) between exon 4 and 5:

**Exon1**

**GGTTTGAGACTGTGTCGGAAGAGGAGAAGGGGGGCAAAGTCTATCAGGTGTTTGAAAGT**

**G--F--E--T--V--S--E--E--E--K--G--G--K--V--Y--Q--V--F--E--S-**

**GTGGCTAAGAAGTATGATGTGATGAATGATATGATGAGTCTTGGTATCCATCGTGTTTGG**

**-V--A--K--K--Y--D--V--M--N--D--M--M--S--L--G--I--H--R--V--W-**

**Exon2**

**AAGGATTTGCTGCTCTGGAAGATGCACCCGCTTCCTGGGACCCAGCTGCTTGATGTTGCT**

**-K--D--L--L--L--W--K--M--H--P--L--P--G--T--Q--L--L--D--V--A-**

**GGAGGCACAGGTGACATTGCATTCCGGTTCCTTAATTATGTTCAGTCCCAGCATCAGAGA**

**-G--G--T--G--D--I--A--F--R--F--L--N--Y--V--Q--S--Q--H--Q--R-**

**Exon3**

**AAACAGAAGAGGCAGTTAAGGGCCCAACAAAATTTATCCTGGGAAGAAATTGCCAAAGAG**

**-K--Q--K--R--Q--L--R--A--Q--Q--N--L--S--W--E--E--I--A--K--E-**

**TACCAGAATGAAGAAGATTCCTTGGGCGGGTCTCGTGTCGTGGTGTGTGACATCAACAAG**

**-Y--Q--N--E--E--D--S--L--G--G--S--R--V--V--V--C--D--I--N--K-**

**GAGATGCTAAAGGTTGGAAAGCAGAAAGCCTTGGCTCAAGGATACAGAGCTGGACTTGCA**

**-E--M--L--K--V--G--K--Q--K--A--L--A--Q--G--Y--R--A--G--L--A-**

**TGGGTATTAGGAGATGCTGAAGAACTGCCCTTTGATGATGACAAGTTTGATATTTACACC**

**-W--V--L--G--D--A--E--E--L--P--F--D--D--D--K--F--D--I--Y--T-**

**Exon4 *Fragment of Intron4 (25bp)***

**ATTGCCTTTGGGATCCGGAATGTCACACACATTGATCAG*atatgaatggctgcatcctca***

**-I--A--F--G--I--R--N--V--T--H--I--D--Q--I--*--**

***gctt*GCACTCCAGGAAGCTCATCGGGTGCTGAAACCAGGAGGACGGTTTCTCTGTCTGGA**

**Exon5**

**ATTTAGCCAAGTGAACAATCCCCTCATATCCAGGCTTTATGATCTATATAGCTTCCAGGT**

**Exon6**

**CATCCCTGTCCTGGGAGAGGTCATCGCTGGAGACTGGAAGTCCTATCAGTACCTTGTAGA**

**Exon7**

**GAGTATCCGAAGGTTTCCGTCTCAGGAAGAGTTCAAGGACA**

**Band 735 bp** – cloned and sequenced fragment of *COQ5* mRNA same in length and exon composition as reference **WT sequence**:

**Exon1**

**GGGTTTGAGACTGTGTCGGAAGAGGAGAAGGGGGGCAAAGTCTATCAGGTGTTTGAAAGT**

**-G--F--E--T--V--S--E--E--E--K--G--G--K--V--Y--Q--V--F--E--S-**

**GTGGCTAAGAAGTATGATGTGATGAATGATATGATGAGTCTTGGTATCCATCGTGTTTGG**

**-V--A--K--K--Y--D--V--M--N--D--M--M--S--L--G--I--H--R--V--W-**

**Exon2**

**AAGGATTTGCTGCTCTGGAAGATGCACCCGCTTCCTGGGACCCAGCTGCTTGATGTTGCT**

**-K--D--L--L--L--W--K--M--H--P--L--P--G--T--Q--L--L--D--V--A-**

**GGAGGCACAGGTGACATTGCATTCCGGTTCCTTAATTATGTTCAGTCCCAGCATCAGAGA**

**-G--G--T--G--D--I--A--F--R--F--L--N--Y--V--Q--S--Q--H--Q--R-**

**Exon3**

**AAACAGAAGAGGCAGTTAAGGGCCCAACAAAATTTATCCTGGGAAGAAATTGCCAAAGAG**

**-K--Q--K--R--Q--L--R--A--Q--Q--N--L--S--W--E--E--I--A--K--E-**

**TACCAGAATGAAGAAGATTCCTTGGGCGGGTCTCGTGTCGTGGTGTGTGACATCAACAAG**

**-Y--Q--N--E--E--D--S--L--G--G--S--R--V--V--V--C--D--I--N--K-**

**GAGATGCTAAAGGTTGGAAAGCAGAAAGCCTTGGCTCAAGGATACAGAGCTGGACTTGCA**

**-E--M--L--K--V--G--K--Q--K--A--L--A--Q--G--Y--R--A--G--L--A-**

**TGGGTATTAGGAGATGCTGAAGAACTGCCCTTTGATGATGACAAGTTTGATATTTACACC**

**-W--V--L--G--D--A--E--E--L--P--F--D--D--D--K--F--D--I--Y--T-**

**Exon4**

**ATTGCCTTTGGGATCCGGAATGTCACACACATTGATCAGGCACTCCAGGAAGCTCATCGG**

**-I--A--F--G--I--R--N--V--T--H--I--D--Q--A--L--Q--E--A--H--R-**

**Exon5**

**GTGCTGAAACCAGGAGGACGGTTTCTCTGTCTGGAATTTAGCCAAGTGAACAATCCCCTC**

**-V--L--K--P--G--G--R--F--L--C--L--E--F--S--Q--V--N--N--P--L-**

**ATATCCAGGCTTTATGATCTATATAGCTTCCAGGTCATCCCTGTCCTGGGAGAGGTCATC**

**-I--S--R--L--Y--D--L--Y--S--F--Q--V--I--P--V--L--G--E--V--I-**

**Exon6**

**GCTGGAGACTGGAAGTCCTATCAGTACCTTGTAGAGAGTATCCGAAGGTTTCCGTCTCAG**

**-A--G--D--W--K--S--Y--Q--Y--L--V--E--S--I--R--R--F--P--S--Q-**

**Exon7**

**GAAGAGTTCAAGGACA**

**-E--E--F--K--D—**

**Band 628 bp** – cloned and sequenced fragment of *COQ5* mRNA with missing **Exon4**:

**Exon 1**

**GGGTTTGAGACTGTGTCGGAAGAGGAGAAGGGGGGCAAAGTCTATCAGGTGTTTGAAAGT**

**-G--F--E--T--V--S--E--E--E--K--G--G--K--V--Y--Q--V--F--E--S-**

**GTGGCTAAGAAGTATGATGTGATGAATGATATGATGAGTCTTGGTATCCATCGTGTTTGG**

**-V--A--K--K--Y--D--V--M--N--D--M--M--S--L--G--I--H--R--V--W-**

**Exon 2**

**AAGGATTTGCTGCTCTGGAAGATGCACCCGCTTCCTGGGACCCAGCTGCTTGATGTTGCT**

**-K--D--L--L--L--W--K--M--H--P--L--P--G--T--Q--L--L--D--V--A-**

**GGAGGCACAGGTGACATTGCATTCCGGTTCCTTAATTATGTTCAGTCCCAGCATCAGAGA**

**-G--G--T--G--D--I--A--F--R--F--L--N--Y--V--Q--S--Q--H--Q--R-**

**Exon 3**

**AAACAGAAGAGGCAGTTAAGGGCCCAACAAAATTTATCCTGGGAAGAAATTGCCAAAGAG**

**-K--Q--K--R--Q--L--R--A--Q--Q--N--L--S--W--E--E--I--A--K--E-**

**TACCAGAATGAAGAAGATTCCTTGGGCGGGTCTCGTGTCGTGGTGTGTGACATCAACAAG**

**-Y--Q--N--E--E--D--S--L--G--G--S--R--V--V--V--C--D--I--N--K-**

**GAGATGCTAAAGGTTGGAAAGCAGAAAGCCTTGGCTCAAGGATACAGAGCTGGCACTCCA**

**-E--M--L--K--V--G--K--Q--K--A--L--A--Q--G--Y--R--A--G--T--P-**

**Exon 5**

**GGAAGCTCATCGGGTGCTGAAACCAGGAGGACGGTTTCTCTGTCTGGAATTTAGCCAAGT**

**-G--S--S--S--G--A--E--T--R--R--T--V--S--L--S--G--I--*--P--S-**

**GAACAATCCCCTCATATCCAGGCTTTATGATCTATATAGCTTCCAGGTCATCCCTGTCCT**

**-E--Q--S--P--H--I--Q--A--L--*--S--I--*--L--P--G--H--P--C--P-**

**Exon 6**

**GGGAGAGGTCATCGCTGGAGACTGGAAGTCCTATCAGTACCTTGTAGAGAGTATCCGAAG**

**-G--R--G--H--R--W--R--L--E--V--L--S--V--P--C--R--E--Y--P--K-**

**Exon 7**

**GTTTCCGTCTCAGGAAGAGTTCAAGGACAT**

**-V--S--V--S--G--R--V--Q--G--H-**

**Band 585 bp** – cloned and sequenced fragment of *COQ5* mRNA with missing **Exon2:**

**Exon1**

**GGGTTTGAGACTGTGTCGGAAGAGGAGAAGGGGGGCAAAGGTGACATTGCATTCCGGTTC**

**-G--F--E--T--V--S--E--E--E--K--G--G--K--G--D--I--A--F--R--F-**

**CTTAATTATGTTCAGTCCCAGCATCAGAGAAAACAGAAGAGGCAGTTAAGGGCCCAACAA**

**-L--N--Y--V--Q--S--Q--H--Q--R--K--Q--K--R--Q--L--R--A--Q--Q-**

**Exon3**

**AATTTATCCTGGGAAGAAATTGCCAAAGAGTACCAGAATGAAGAAGATTCCTTGGGCGGG**

**-N--L--S--W--E--E--I--A--K--E--Y--Q--N--E--E--D--S--L--G--G-**

**TCTCGTGTCGTGGTGTGTGACATCAACAAGGAGATGCTAAAGGTTGGAAAGCAGAAAGCC**

**-S--R--V--V--V--C--D--I--N--K--E--M--L--K--V--G--K--Q--K--A-**

**TTGGCTCAAGGATACAGAGCTGGACTTGCATGGGTATTAGGAGATGCTGAAGAACTGCCC**

**-L--A--Q--G--Y--R--A--G--L--A--W--V--L--G--D--A--E--E--L--P-**

**Exon4**

**TTTGATGATGACAAGTTTGATATTTACACCATTGCCTTTGGGATCCGGAATGTCACACAC**

**-F--D--D--D--K--F--D--I--Y--T--I--A--F--G--I--R--N--V--T--H-**

**ATTGATCAGGCACTCCAGGAAGCTCATCGGGTGCTGAAACCAGGAGGACGGTTTCTCTGT**

**-I--D--Q--A--L--Q--E--A--H--R--V--L--K--P--G--G--R--F--L--C-**

**Exon5**

**CTGGAATTTAGCCAAGTGAACAATCCCCTCATATCCAGACTTTATGATCTATATAGCTTC**

**-L--E--F--S--Q--V--N--N--P--L--I--S--R--L--Y--D--L--Y--S--F-**

**Exon6**

**CAGGTCATCCCTGTCCTGGGAGAGGTCATCGCTGGAGACTGGAAGTCCTATCAGTACCTT**

**-Q--V--I--P--V--L--G--E--V--I--A--G--D--W--K--S--Y--Q--Y--L-**

**Exon7**

**GTAGAGAGTATCCGAAGGTTTCCGTCTCAGGAAGAGTTCAAGGACA**

**-V--E--S--I--R--R--F--P--S--Q--E--E--F--K--D--**

**Band 539** - cloned and sequenced fragment of *COQ5* mRNA with missing **Exon4** and **Exon5:**

**Exon1**

**GGGTTTGAGACTGTGTCGGAAGAGGAGAAGGGGGGCAAAGTCTATCAGGTGTTTGAAAGT**

**-G--F--E--T--V--S--E--E--E--K--G--G--K--V--Y--Q--V--F--E--S-**

**GTGGCTAAGAAGTATGATGTGATGAATGATATGATGAGTCTTGGTATCCATCGTGTTTGG**

**-V--A--K--K--Y--D--V--M--N--D--M--M--S--L--G--I--H--R--V--W-**

**Exon2**

**AAGGATTTGCTGCTCTGGAAGATGCACCCGCTTCCTGGGACCCAGCTGCTTGATGTTGCT**

**-K--D--L--L--L--W--K--M--H--P--L--P--G--T--Q--L--L--D--V--A-**

**GGAGGCACAGGTGACATTGCATTCCGGTTCCTTAATTATGTTCAGTCCCAGCATCAGAGA**

**-G--G--T--G--D--I--A--F--R--F--L--N--Y--V--Q--S--Q--H--Q--R-**

**Exon3**

**AAACAGAAGAGGCAGTTAAGGGCCCAACAAAATTTATCCTGGGAAGAAATTGCCAAAGAG**

**-K--Q--K--R--Q--L--R--A--Q--Q--N--L--S--W--E--E--I--A--K--E-**

**TACCAGAATGAAGAAGATTCCTTGGGCGGGTCTCGTGTCGTGGTGTGTGACATCAACAAG**

**-Y--Q--N--E--E--D--S--L--G--G--S--R--V--V--V--C--D--I--N--K-**

**GAGATGCTAAAGGTTGGAAAGCAGAAAGCCTTGGCTCAAGGATACAGAGCTGGCTTTATG**

**-E--M--L--K--V--G--K--Q--K--A--L--A--Q--G--Y--R--A--G--F--M-**

**Exon6**

**ATCTATATAGCTTCCAGGTCATCCCTGTCCTGGGAGAGGTCATCGCTGGAGACTGGAAGT**

**-I--Y--I--A--S--R--S--S--L--S--W--E--R--S--S--L--E--T--G--S-**

**Exon7**

**CCTATCAGTACCTTGTAGAGAGTATCCGAAGGTTTCCGTCTCAGGAAGAGTTCAAGGACA**

**-P--I--S--T--L--*--R--V--S--E--G--F--R--L--R--K--S--S--R--T-**

Supplementary Figure 3. Detailed presentation of cloned fragments of cDNA of Patient 5 and her family.

9. Li H, Durbin R. Fast and accurate short read alignment with Burrows-Wheeler transform. Bioinformatics. 2009;25(14):1754-1760.

10. McKenna A, Hanna M, Banks E, et al. The Genome Analysis Toolkit: a MapReduce framework for analyzing next-generation DNA sequencing data. Genome Res. 2010;20(9):1297-1303.

11. Andrews, S. (2010). FastQC: A Quality Control Tool for High Throughput Sequence Data [Online]. Available online at: http://www.bioinformatics.babraham.ac.uk/projects/fastqc/

12. Danecek P, Bonfield JK, Liddle J, et al. Twelve years of SAMtools and BCFtools. Gigascience. 2021;10(2):giab008.

13. García-Alcalde F, Okonechnikov K, Carbonell J, et al. Qualimap: evaluating next-generation sequencing alignment data. Bioinformatics. 2012;28(20):2678-2679.

14. Ewels P, Magnusson M, Lundin S, Käller M. MultiQC: summarize analysis results for multiple tools and samples in a single report. Bioinformatics. 2016;32(19):3047-3048.

15. Tan A, Abecasis GR, Kang HM. Unified representation of genetic variants. Bioinformatics. 2015;31(13):2202-2204.

16. McLaren W, Gil L, Hunt SE, et al. The Ensembl Variant Effect Predictor. Genome Biol. 2016;17(1):122.

17. Karczewski KJ, Francioli LC, Tiao G, et al. The mutational constraint spectrum quantified from variation in 141,456 humans. Nature. 2020;581(7809):434-443.

18. Amberger JS, Bocchini CA, Schiettecatte F, Scott AF, Hamosh A. OMIM.org: Online Mendelian Inheritance in Man (OMIM®), an online catalog of human genes and genetic disorders. Nucleic Acids Res. 2015;43(Database issue):D789-D798.

19. Landrum MJ, Lee JM, Benson M, et al. ClinVar: improving access to variant interpretations and supporting evidence. Nucleic Acids Res. 2018;46(D1):D1062-D1067.

20. Liu X, Li C, Mou C, Dong Y, Tu Y. dbNSFP v4: a comprehensive database of transcript-specific functional predictions and annotations for human nonsynonymous and splice-site SNVs. Genome Med. 2020;12(1):103.

21. Jaganathan K, Kyriazopoulou Panagiotopoulou S, McRae JF, et al. Predicting Splicing from Primary Sequence with Deep Learning. Cell. 2019;176(3):535-548.e24.

22. Köhler S, Gargano M, Matentzoglu N, et al. The Human Phenotype Ontology in 2021. Nucleic Acids Res. 2021;49(D1):D1207-D1217.

23. Li MM, Datto M, Duncavage EJ, et al. Standards and Guidelines for the Interpretation and Reporting of Sequence Variants in Cancer: A Joint Consensus Recommendation of the Association for Molecular Pathology, American Society of Clinical Oncology, and College of American Pathologists. J Mol Diagn. 2017;19(1):4-23.
